# Underwater Acoustic Target Recognition Based on Attention Residual Network

**DOI:** 10.3390/e24111657

**Published:** 2022-11-15

**Authors:** Juan Li, Baoxiang Wang, Xuerong Cui, Shibao Li, Jianhang Liu

**Affiliations:** 1 College of Computer Science and Technology, China University of Petroleum (East China), Qingdao 266580, China; 2 College of Oceanography and Space Informatics, China University of Petroleum (East China), Qingdao 266580, China

**Keywords:** underwater acoustics, combined features, channel attention module, ResNet, transfer learning

## Abstract

Underwater acoustic target recognition is very complex due to the lack of labeled data sets, the complexity of the marine environment, and the interference of background noise. In order to enhance it, we propose an attention-based residual network recognition method (AResnet). The method can be used to identify ship-radiated noise in different environments. Firstly, a residual network is used to extract the deep abstract features of three-dimensional fusion features, and then a channel attention module is used to enhance different channels. Finally, the features are classified by the joint supervision of cross-entropy and central loss functions. At the same time, for the recognition of ship-radiated noise in other environments, we use the pre-training network AResnet to extract the deep acoustic features and apply the network structure to underwater acoustic target recognition after fine-tuning. The two sets of ship radiation noise datasets are verified, the DeepShip dataset is trained and verified, and the average recognition accuracy is 99%. Then, the trained AResnet structure is fine-tuned and applied to the ShipsEar dataset. The average recognition accuracy is 98%, which is better than the comparison method.

## 1. Introduction

In recent years, underwater acoustic target recognition has been widely used to detect marine ships, evaluate the impact of ship radiated noise, and recognize marine life [1]. However, feature identification of underwater acoustic targets has become difficult due to the time-varying nature of underwater acoustic channels, the correlated absorption and scattering of sound, and the increasingly complex marine noise environment. Usually, underwater acoustic target recognition is performed by well-trained sonar specialists. The identification results are unstable due to the different experiences of the specialists and lousy weather [2]. Therefore, using an underwater acoustic target recognition algorithm to identify ship radiated noise becomes particularly important. 

Thanks to the development of deep learning methods, the accuracy of recognition and recognition algorithms in the field of audio signal processing has improved significantly. The deep learning method is used to process massive amounts of data, extracting more useful sample features for recognition and showing good performance in underwater acoustic target recognition [3,4]. Li et al. [5] introduced slope entropy into underwater acoustic signal processing to obtain higher recognition rate. Hong et al. [6] used fused features to train an 18-layer residual neural network containing the central loss function of the embedding layer (namely ResNet18 in this paper) and adopted various strategies to prevent model overfitting, which improved the accuracy to 94.3% on the ShipsEar dataset. Hong et al. [6] used a joint loss function containing a central loss function to monitor the characteristics of different underwater acoustic targets. However, using the joint loss function will equally monitor all features, including ocean background noise and other interference information, reducing the network’s recognition effect. At the same time, for the identification of data in different environments, the number of channels required is different, the number of channels is small, and the features of each dimension cannot be fully extracted, while the number of channels is large, which will contain more ocean background noise and other interference information, which will affect the recognition effect. In addition, when ResNet18 is applied to other ship-radiated noise datasets, it may lead to overfitting problems due to the small number of samples available for training.

The attention mechanism has made gratifying progress in solving the problem that residual networks are affected by interference information. Chen et al. [7] use reverse attention to guide the side output residual learning in a top-down manner, which improves the residual network’s attention to residual details and improves the detection performance. Lu et al. [8] used the three-layer parallel residual network structure to learn the spectrum and spatial features, and then used the three-dimensional attention module to enhance the expressiveness of the features from the channel and spatial domain, achieving better classification results. Fan et al. [9] use the trunk branch of the residual structure to extract features, and mask branches imitate the attention mechanism to add soft weights to the features extracted from the trunk branch to optimize the extracted features and obtain better performance. Tripathi and Mishra [10] used four two-layer residual blocks to build the network. After the fourth layer, they used attention modules to deal with intra-class inconsistencies, which improved the compactness and increased by 11.50% and 19.50%, respectively, over the benchmark model on the two datasets. 

Inspired by the success of the attention mechanism and overcoming the problems of residual networks, we propose a new recognition method based on attention residual networks (AResnet). We use a three-layer residual block. The trunk branch of the residual block uses a 7 × 7 convolution kernel, and the mask branch uses a 1 × 1 convolution kernel. Each mask branch is followed by a spatial attention module to weight different channels. In order to adapt the network to different environments, we use residual blocks with 256 channels in three layers and 512 channels in two layers to fully extract features. After each residual block, we use channel attention module to suppress ocean background noise and other interference information and enhance features that can better represent target features.

There is also some literature that introduces attention mechanisms into underwater acoustic target recognition. Xiao et al. [11] placed the attention module in front of the hidden layer of DNN, and only retained the features related to the target to suppress environmental noise and marine ship interference, thus achieving high accuracy of target detection and recognition. Hu et al. [12] used the depth direction separable convolution filter to decompose the original time-domain ship radiated noise signal into different frequency components, and then extracted the signal features based on auditory perception. Deep functions are integrated into the integration layer. Time-extended convolution is used for long-term context modeling. Liu et al. [13] used the multi-resolution pooled convolution scheme based on the Inception model to build the MCNN architecture, then used the first eight layers of the ResNet50 model to extract features, and finally used the location attention module and spatial attention module to optimize the feature information in parallel. The average recognition accuracy on ShipsEar was 95.6%. Yang et al. [14] designed a set of multi-scale depth convolution filters to decompose the original time domain signal into signals with different frequency components, which realized the effective classification of ship-radiated noise. Xue et al. [15] used a set of one-dimensional convolutions to decompose the acoustic signal into the basic signal and used two two-layer residual blocks to extract the basic signal features. After the residual block, the channel attention mechanism was added to enhance the energy of residual convolution stable spectrum features and obtain better recognition results. 

We also introduce an attention mechanism into underwater acoustic target recognition. Different from the above literature, we have constructed a new residual block, the attention-based convolution residual block, in which the main branch of the residual block has three layers of convolution, the size of the convolution core is 7 × 7, and the mask branch uses 1 × 1 convolution core, and then uses the channel attention module to assign weights to different channels of the mask branch. At the same time, in order to make the network adapt to the recognition of different environments, we use three 256-channel residual blocks and two 512-channel residual blocks to fully extract target features by increasing the number of channels of the attention-based residual blocks. At the same time, in order to reduce the interference of ocean background noise and multi-target noise, a channel attention module is used after each residual block to reduce the feature weight of ocean background noise and multi-target noise, enhance the feature weight of the target, and improve the recognition effect. Finally, the joint loss function, including the cross-entropy loss function and the center loss function, can differentially supervise the abstract features of different channels, effectively suppress the ocean background noise, and improve the adaptability of AResnet in different environments. The network can fully extract the features of different dimensions of underwater acoustic targets and adaptively enhance the abstract features of different channels. The joint loss function is used to differentially supervise the abstract features of different channels so that the features that can better represent the signal characteristics are concentrated in the class center distribution, effectively improving the recognition effect.

The difficulty and number of samples obtained are different in different environments. Although the early stopping strategy and the method of dynamically adjusting the learning rate are adopted in the model’s design to avoid overfitting, there is still a problem of insufficient samples when applied to the ship radiated noise dataset with a small sample size. For this, we use the idea of transfer learning. First, AResnet is pre-trained on the DeepShip dataset. The network structure is then fine-tuned and applied to the small-sample ship radiated noise dataset. The proposed method is validated on two datasets of different sizes. After training and testing on the DeepShip dataset, the average accuracy on the test set is 99.0%. The pre-trained AResnet is trained and tested on the ShipsEar dataset, and the average accuracy on the test set is 98.0%, which is a 3.6% improvement over the ResNet18 method proposed by Hong et al. [6].

The main contributions of this paper are as follows:

1. The joint loss function, including the center loss function and cross-entropy loss function, combined with the channel attention module, improves the problem of equal supervision of all features when mining discriminative information of fused features. These features will contain other interfering information, such as ocean background noise, and the same supervision will reduce the recognition effect of the model.
2. The proposed AResnet method enhances different channels by increasing the width of the residual network and combining the channel attention module, which not only meets the requirements of network width due to environmental changes but also suppresses background noise interference.
3. Aiming at the problem of insufficient samples, when the AResnet method is applied to the small sample ship radiated noise data set, the trained network parameters are migrated, and the structure is adjusted, which greatly improves the recognition effect of ship radiated noise collected in different environments.
4. The proposed AResnet model is validated on datasets of different environments and scales. The results are better than other methods.

The rest of this article is organized as follows. Section 2 describes the feature extraction method and the proposed method for recognition. Section 3 presents the experimental data and preprocessing methods, while Section 4 presents the recognition results and analysis. Finally, Section 5 concludes the paper.

## 2. Method

**Figure 1:**
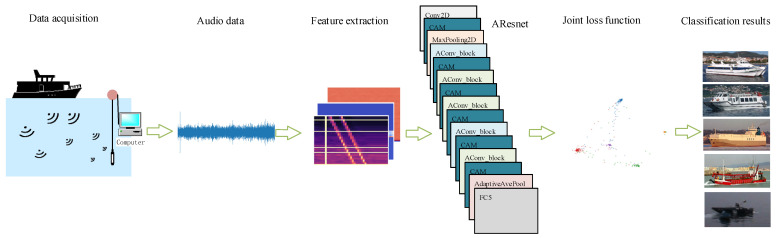
Architecture diagram recognition of the recognition methods based on attentional residual neural networks.

The proposed recognition method mainly includes feature extraction and classifier design. In terms of feature extraction, various feature extraction algorithms are used to extract features from each input signal frame. Then, multiple features are fused into three-dimensional fusion features. The SpecAugment data augmentation method is used to augment the three-dimensional fused features and take them as input to the AResnet network. AResnet includes multiple attention-based convolutional residual blocks (AConv_block) and channel attention modules (CAM). AConv_block is used to extract deep abstract features of ship-radiated noise. CAM performs differential enhancement on different channels so that the extracted abstract features can better represent underwater acoustic targets. Finally, we use a joint loss function, including a center loss function and a cross-entropy loss function, to perform differential supervision on the features of different channels to achieve underwater acoustic target recognition. The architecture diagram is shown in Figure 1.

### 2.1. Feature Extraction

It is necessary to extract some important features from each audio signal frame to train AResnet. The original underwater acoustic signal is highly nonstationary but can be regarded as local stationary. The signal with a sampling frequency of 22,050 Hz is divided into frames with a window length of 43 ms, and each frame has 512 sampling points. In order to ensure local stability, 40 frames are selected to extract underwater acoustic signal features. Generally speaking, underwater acoustic signal features extracted by different methods have different signal characterization capabilities, and using multiple features for fusion can obtain better recognition results [16]. In order to represent the underwater acoustic signal more, the Log-Mel spectrum, Mel Frequency Cepstrum Coefficient, Contrast, Chroma, Tonnetz, and Zero crossing rate are extracted and fused into three-dimensional fusion features.

**Figure 2:**
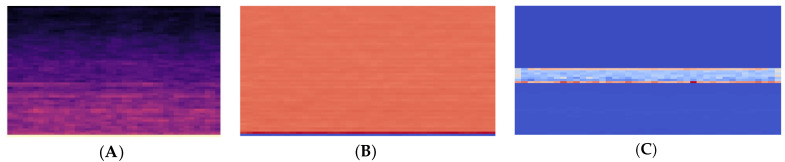
Visualize extracted features. By the model, from the left (**A**) Log-Mel spectrogram (**B**) Mel Frequency Cepstral Coefficients (**C**) features after splicing of Chroma, Contrast, Tonnetz, and zero-crossing rate.

Log-Mel spectrum—The spectrum diagram can intuitively display the local frequency information of the input signal, which has more characteristics than the original audio signal. However, the sound level heard by human ears does not have a linear relationship with the actual (Hz) frequency, and the spectrum of linear scale is not enough to simulate the auditory characteristics of human ears. Mel frequency is more in line with the auditory characteristics of human ears. The Mel spectrum as the network’s input can highlight the spectrum characteristics of underwater acoustic signals and obtain better recognition results. The formula for converting from frequency to Mel scale is:

$$M\left(f\right)=1125\;\log\left(1+\frac{f}{700}\right),$$

where *f* represents a linear frequency. Sixty Mel filter banks are considered to convert the linear spectrum into the Mel spectrum. Then, the Mel spectrum is logarithmically transformed into a Log-Mel spectrum. The Log-Mel spectrum is shown in Figure 2A. 

MFCC—The Mel-Frequency Cepstral Coefficients (MFCCs) are other features extracted by applying a Discrete Cosine Transform (DCT) to the log-compressed Mel scale power spectrum [17]. MFCC helps to remove background noise from the recording and obtain effective target features in the spectrum. The MFCC is shown in Figure 2B.

Contrast—Contrast is evaluated by dividing the spectrogram into different sub-bands [18]. For each sub-band, the energy contrast is estimated by comparing the average energy of the top and bottom quantiles. The high contrast values generally correspond to clear narrowband signals, and low contrast values correspond to broadband noise. We consider six coefficients per frame for this feature.

Chroma—Chroma represents the tonal content of an audio signal in a compact form and shows how much energy is present at each tone level [19]. We consider 24 Chroma per frame.

Tonnetz—Tonnetz obtained 12 chromaticity vectors by mapping pitches onto the vertices of this hexahedral polyhedron, converting the harmonic relations to small Euclidean distances [20].

Zero-crossing rate—The zero-crossing rates are the number of times the signal crosses the zero point per frame.

The Contrast, Chroma, Tonnetz, and zero-crossing rates are combined in a concatenated form. The concatenated features are shown in Figure 2C. In feature fusion, the Log-Mel spectrum is used as the first channel, the MFCC is used as the second channel, and the cascade features of Contrast, Chroma, Tonnetz, and zero-crossing rate are used as the third channel. As the network input, the dimension of this three-dimensional fusion feature is 3 × 60 × 41.

**Figure 3:**
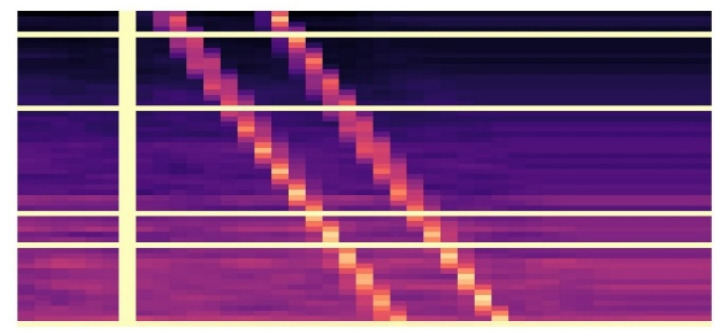
Log-Mel spectrogram after data augmentation.

In order to avoid model overfitting, after feature fusion, the fused features are enhanced. When other dimensional features remain unchanged, SpecAugment [21] is used to enhance the log Mel spectrum before training to avoid overfitting. SpecAugment originates from the data enhancement method of speech recognition and can be directly applied to the feature input of neural networks. The enhancement strategy includes distortion features, masking frequency channel blocks, and masking time blocks [21]. We perform multiple distortion features, mask frequency channel block, and mask time block operations on the Log-Mel spectrogram of the first channel of the fused feature. The log Mel spectrum features after data enhancement are shown in Figure 3.

### 2.2. Attention-Based Residual Network

**Figure 4:**
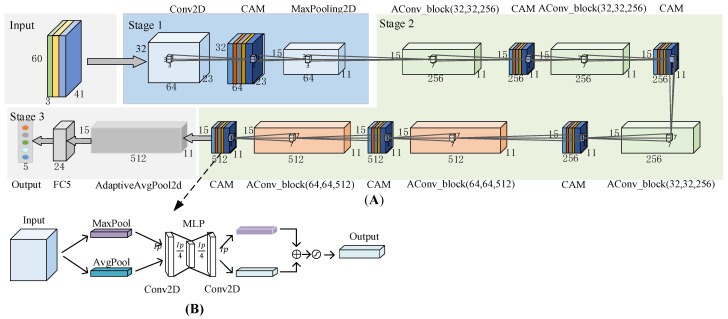
Overall architecture of the attention-based residual neural network. By the model, from the top (**A**) structure of AResnet (**B**) structure of CAM.

The attention-based residual network proposed in this paper is composed of AConv_block and CAM. The role of CAM is to differentially enhance the abstract features of different channels in the network, and its structure is shown in Figure 4B. After the data passes through the adaptive max-pooling layer and the adaptive average pooling layer, data are entered into the multi-layer perceptron. The results are added together using the SoftMax activation function to output the probability of each class. Each convolutional layer uses 3 × 3 filter kernels with Ip/4 and Ip filters in each subsequent layer. The network applies a Rectified Linear Unit (ReLU) nonlinear activation function to the output of the first convolutional layer.

The details are listed as follows:

Stage 1: The fused features with the size of 3 × 60 × 41 are zero-padding and input into a convolutional layer with 3 × 3 filter kernels and a stride of 2 × 2. After the batch normalization layer, the Gaussian Error Linear Unit (GELU) non-linear activation function is applied to their outputs. After CAM and max-pooling layer, the shape is adjusted to 64 × 32 × 23. The GELU is defined as:

$$\mathrm{GELU}\left(x_{i}\right)=0.5\times x_{i}\left(1+\tan h\left(\sqrt{\frac{2}{\pi }}\left(x_{i}+0.044715\times x_{i}{}^{3}\right)\right)\right),$$

where $x_{i}$ is the input to the nonlinear activation on the *i*th channel.

**Figure 5:**
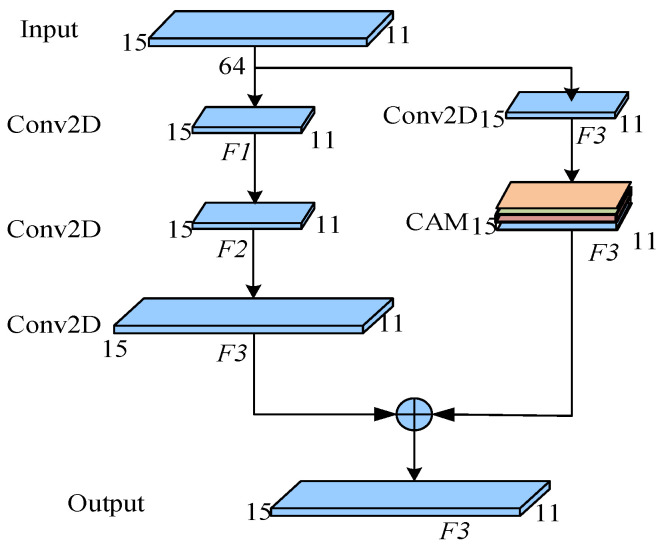
The structure of the AConv_block (*F*1, *F*2, *F*3), where the *F*1, *F*2, and *F*3 represent the number of convolutional layers.

Stage 2: The network is stacked by AConv_block (*F*1, *F*2, *F*3) and CAM. The AConv_block (*F*1, *F*2, *F*3) is shown in Figure 5, where *F*1, *F*2, and *F*3 are the number of convolutional layers. Before the convolution operation of the main branch, the input is filled with 3 × 3 Zero fill, then enter with 7 × 7 Convolution layer of filter core. The convolution layer of mask branch uses 1 × 1 Filter core. 

Stage 3: After the adaptive average pooling layer, the shape is adjusted from 512 × 15 × 11 to 512 × 7 × 5. After the data are flattened, they are input into a fully connected layer consisting of 24 hidden units. Finally, the fully connected layer with 5 hidden units is connected.

It should be mentioned that to increase the model’s generalization ability, AConv_block (*F*1, *F*2, *F*3) uses GELU after the convolution operation. 

### 2.3. Joint Loss Function

The cross-entropy loss function is a supervisory signal to train the network in most convolutional neural networks. However, the problem with using the cross-entropy loss function is that the intra-class variation of sample features is noticeable, reducing the model’s discriminative ability. Wen et al. [22] proposed a solution that uses the center loss function to compensate for the cross-entropy loss function, which can enhance the robustness and discriminative ability of the model through the joint supervision of the cross-entropy and center loss function. Specifically, the center loss function provides a center for each class, and the sample features of the same class are distributed around the center of the class. The joint loss function, which includes the central loss function, minimizes the intra-class distance while keeping the feature separability, which makes the intra-class distance closer and the inter-class distance farther. The recognition task ${\left\{\left(x_{i},y_{i}\right)\right\}}_{i=1}^{N}$ contains N samples of $x_{i}$ and their corresponding labels $y_{i}$. The samples are entered into the network to extract the sample features $F\left(x_{i}\right)$.The joint loss function is defined as:

$$L_{c}\left(class=j\right)-\ln\left(\frac{e^{w_{j}^{T}x+b}}{\sum_{i=1}^{N}e^{w_{j}^{T}x+b}}\right)+\lambda \sum_{i=1}^{N}D\left(f\left(x_{i}\right),c_{i}\right),$$

where the first term represents the cross-entropy loss function, the second term represents the center loss function, and *λ* ∈ [0, 1] is used to balance the two-loss functions. $c_{i}$ is the class center of the mini-batch dataset, the function D(·) represents the distance function, j is the class number, x is the input, $w_{j}$ is the weight, b is the bias, and N is the total number of classes. The center loss function will adjust the distance between samples of the same class to focus on the class center during training.

**Figure 6:**
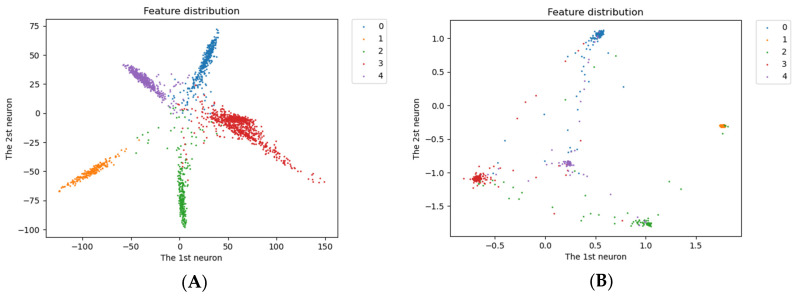
The effect of different loss functions will be illustrated using the 2D features of the test dataset. By the model, from the left (**A**) cross-entropy loss function (**B**) joint loss function.

Figure 6 plots the 2D features with different weights obtained when validating on the ShipsEar dataset to illustrate the distribution. Figure 6A shows that under the supervision of the cross-entropy loss function, the data exhibits clear separability, but there is still significant intra-class variation. It is not appropriate to use these features directly for identification. Figure 6B shows that, under the supervision of the joint loss function, the inter-class distance changes significantly, and the feature distribution is concentrated. This is more conducive to the recognition of the classifier.

## 3. Experimental Data and Preprocessing

### 3.1. Experimental Data

The underwater acoustic target datasets based on ship-radiated noise used in the experiments are DeepShip [23] dataset and ShipsEar [24] dataset. 

**Figure 7:**
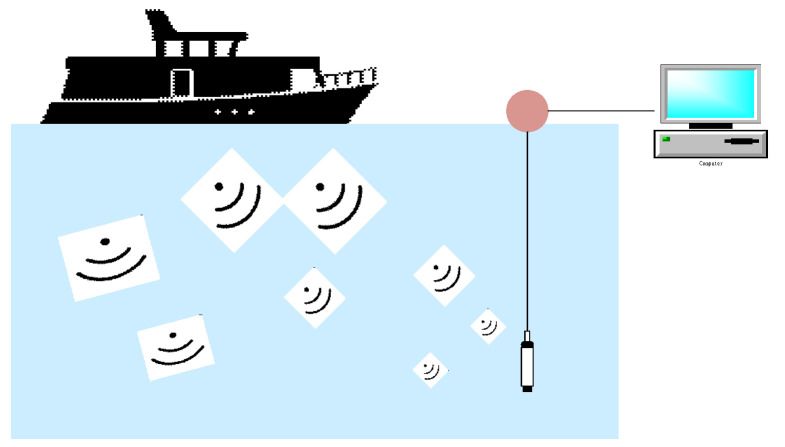
Hydrophone setup for underwater recordings of DeepShip vessel noise.

The Deepship dataset was generated from audio recordings collected at the Georgia Strait Delta node between 2 May 2016, and 4 October 2018. The audio was recorded using an IcListen AF hydrophone with a sampling rate of 32,000 Hz, the acquisition diagram is shown in Figure 7. The hydrophone is placed at a depth of 141–147 below the horizontal plane. The upper part of the hydrophone is connected to the surface buoy. In order to mark the recorded data, the Automatic Identification System (AIS) data are used to obtain the location and time stamp of any particular vessel passing through the deployed sensors. We only consider the signal sent by the ship when there is only one ship within the hydrophone radius of 2 km. The data will stop whenever the ship is 2 km away from the hydrophone. The duration of each recording varied from 6 seconds to 1530 s, and a total of 613 pieces of data were acquired. The collected objects come from 265 ships, divided into four categories: tug, cargo ship, passenger ship, and an oil tanker. The dataset is divided as shown in Table 1.

**Figure 8:**
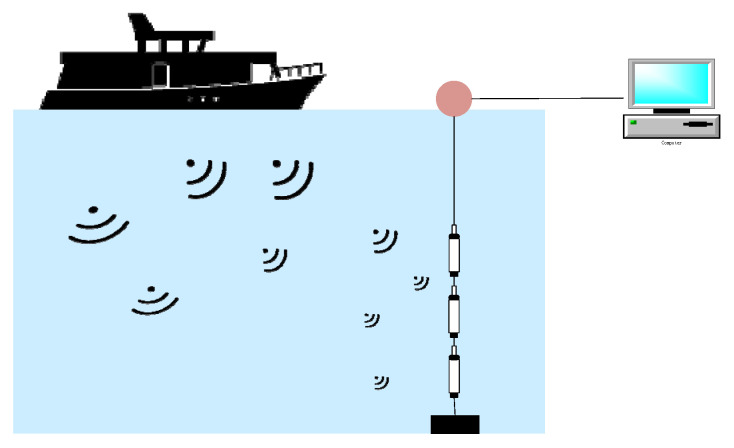
Hydrophone setup for underwater recordings of ShipsEar vessel noise.

The ShipsEar dataset was produced from ship-radiated noise collected along the Spanish Atlantic coast in Northwest Spain. The audio was recorded using a digitalHyd SR-1 recorder with a sampling rate of 52,734 Hz, the acquisition diagram is shown in Figure 8. The upper part of the hydrophone is connected to the surface buoy for easy recovery. The bottom of the hydrophone is connected to the underwater buoy to ensure that it is vertical. The height of the hydrophone on the seabed is selected according to the depth of the mooring point. Whenever possible, three hydrophones with different depths and different gains are used to maximize the dynamic range of the recording. In very shallow areas (below 10 m in depth), record with one or two hydrophones. An auxiliary vessel is used to deploy hydrophones and schedule recording based on vessel movement information obtained from the Port Authority and the Automatic Vessel Identification System (AIS). Each recording lasted from 15 s to 10 min, with a total of 90 records. The collection objects include 11 types of ships, including fishing boats, ocean liners, ferries of various sizes, containers, ro-ro ships, tugboats, pilot boats, yachts, and small sailboats as background noise near the coast. Unlike artificially constructed datasets, real-world data contains more man-made and natural background noise, with high background noise from waves hitting port infrastructure, and occasional sounds from marine mammals, making the experiments more valuable. The ShipsEar dataset is divided into five categories, including four categories of ships and one category of background noise. The detailed division of the dataset is shown in Table 2.

### 3.2. Data Preprocessing

After removing the blank area, the original audio signal is divided into 5-s audio clips. DeepShip obtains 11,174 audio clips, and ShipsEar obtains 2223 audio clips. A total of 70% of the segment is used as the training data set, 20% as the verification data set, and 10% as the test data set. Then, the 5 s audio clip is cut into a sample section containing 20,480 sampling points. Each frame has 512 sample points, and the sample segment includes 40 frames. We can extract signal features from each frame for underwater acoustic target classification. It is worth mentioning that we use the python library Librosa to load audio files and extract the characteristics of ship-radiated noise.

## 4. Experimental Results and Analysis

The AResnet is built using Pytorch 1.8.1 as the backend and verified on a computer with Nvidia GeForce RTX 3060 and AMD R7-5800H CPU to verify the effectiveness of the proposed architecture.

Training used an Adam optimizer with a dynamically adjusted learning rate and joint loss function, including center loss function and cross-entropy loss function as the loss function. The initial value of the learning rate is 0.0001, and the learning rate is dynamically adjusted by decreasing 50% every 10 cycles. The batch size during training is set to 128, the maximum number of the training is set to 300, and the training is performed using the early stopping method. The training stops when the loss on the validation dataset drops below 0.00005 for 20 consecutive epochs. This strategy can effectively reduce training time and avoid overfitting. 

In the demonstration of experimental results, we use precision, recall, and F1-score to evaluate the recognition performance of the network. The formula of each index is as follows:

$$\mathrm{Precision}=\frac{Tp}{Tp+Fp}$$

$$\mathrm{Recall}=\frac{Tp}{Tp+FN}$$

$$F1-\mathrm{score}=\frac{2TP}{2TP+FP+FN}$$

where *TP* is true positive, *FP* is false positive, and *FN* is false negative.

### 4.1. Experimental Results and Analysis of DeepShip Dataset

**Figure 9:**
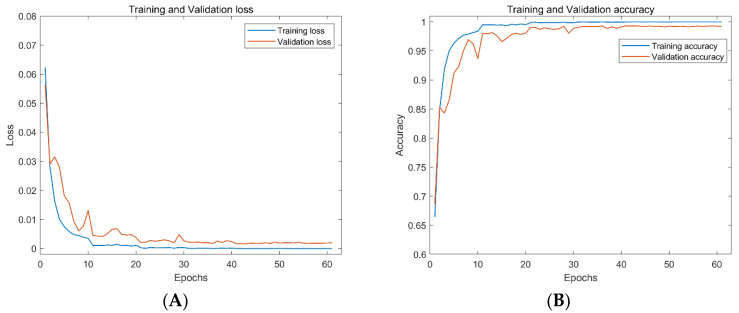
Loss and accuracy of AResnet on the training and test datasets of DeepShip. By the model, from the left (**A**) the loss of the AResnet on the training and test datasets of DeepShip, (**B**) the accuracy of the AResnet on the training and test datasets of DeepShip.

To verify the performance of the architecture proposed in this paper, the DeepShip dataset is used, and the experimental results are shown in Figure 9. The training loss and validation loss decrease rapidly in about 10 cycles. For the convention of comparison, classifier performance is measured using classification accuracy, defined as the average precision. In contrast, the training accuracy and validation datasets increase quickly and soon reach a relatively stable process. The best accuracy on the validation dataset is 99.3%.

Table 3 shows that AResnet achieves an average precision, recall, and F1-score of 99.0% on the four types of ship-radiated noise. Support represents the number of samples for each class on the test dataset. Taking the average accuracy rate as the reference standard, class A (tugboat) has the best recognition effect, with an average accuracy rate of 99.3%. The least effective is class B (cargo ships), with an average accuracy of 98.2%.

The recognition results are compared with DNN, CNN, and CRNN, and the average precision, recall, and F1-score are shown in Table 4. The network is optimized using the Adam optimizer with a learning rate of 0.001. The best network parameters are selected for testing after training 100 times. The structure of DNN is 2048-1024-512-256-128-64-4, in which a Relu activation function follows each layer, and its average precision, recall, and F1-score are 98%. The convolutional layer structure of CNN is 48-128-192-192-128, where each layer is followed by a Relu activation function and uses 3 × 3 convolution kernels to extract features. Zero padding is used to maintain the output size. The convolutional layer of 1, 2, and 5 are followed by the max-pooling layer to compress the output. After the max-pooling layer, a drop of 0.5 is used, and the output result of the fully connected layer with the number of input nodes is 2048-2048-4. Its average precision, recall, and F1-score are all 96.4%. CRNN shows higher performance than DNN and CNN, with 98.6% precision in average precision, recall, and F1-score. The convolutional layer of CRNN uses the structure 64-128-256-256. After each layer, the Relu activation function and the maximum pooling layer are used. The convolutional layers use 3 × 3 convolution kernels to extract features. Two layers of LSTM follow the last convolutional layer for recognition.

These four optimized network structures all show high performance on the DeepShip dataset, but the AResnet proposed in this paper has higher accuracy than other methods.

### 4.2. Experimental Results and Analysis of ShipsEar Dataset

The method proposed in this paper is further tested on the ShipsEar dataset to show the method’s performance in the small sample ship radiated noise dataset. In this work, the ResNet18 is compared with AResnet to verify the effectiveness of the proposed method. ResNet18 used an 18-layer residual network with a central loss function in the embedding layer to train the fused features at an adaptive learning rate. This paper uses the same processing method for data preprocessing and feature extraction for the ShipsEar dataset. We apply the transfer learning idea to data training. The transfer learning framework uses the network trained on the DeepShip dataset to fine-tune the recognition layer of the network model to make it suitable for the recognition of the ShipsEar dataset. The experimental results show that ResNet18 has an accuracy of 94.4% on the test dataset. The accuracy of the attention residual neural network based on transfer learning is 98.0% on the test dataset and is increased by 3.6%.

**Figure 10:**
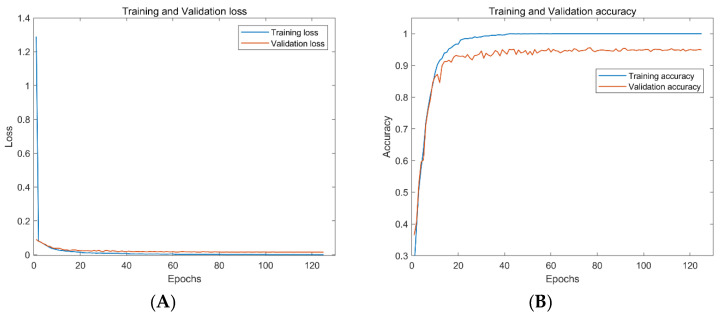
Loss and accuracy of ResNet18 on training and test datasets of ShipsEar. By the model, from the left (**A**) the loss of ResNet18 on the training and test datasets of ShipsEar, (**B**) the accuracy of ResNet18 on the training and test datasets of ShipsEar.

**Figure 11:**
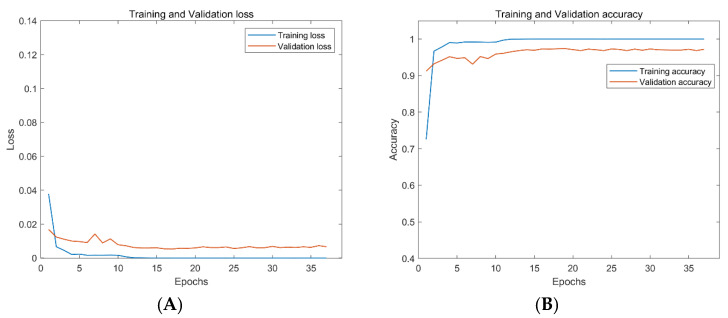
Loss and accuracy of AResnet based on transfer learning on training and test datasets of ShipsEar. By the model, from the left, (**A**) the loss of AResnet based on transfer learning on the training and test datasets of ShipsEar, (**B**) the accuracy of AResnet based on transfer learning on the training and test datasets of ShipsEar.

As can be seen from Figure 10 and Figure 11, both networks show good performance in preventing overfitting. However, the ResNet18 takes 125 epochs to train, and the best accuracy on the validation dataset is 95.4%. The AResnet only needs 37 epochs, and the best accuracy of the validation dataset is 97.4%. The AResnet has shorter training epochs and higher accuracy compared with ResNet18. This proves that the proposed attention-based residual network performs better on passive underwater acoustic target recognition.

Table 5 describes the details of the two recognition models in terms of precision, recall, and F1-score. The average precision, recall, and F1-score of ResNet18 is 94.4%, and support represents the number of each category on the test dataset. The AResnet has an average precision, recall, and F1-score of 98.0%. AResnet outperforms ResNet18 by the classifier performance validated on the test dataset. To further compare the recognition effect of each category, the average precision is uniformly selected for measurement. In ResNet18, the best results are class B (natural environment noise), with an average accuracy of 98.3%. The worst results are class C (motorboats, sailboats, and pilot boats), with an average accuracy of 90.9%. The best and worst recognition results on the test dataset in AResnet are also the average accuracy of 100% and 96.6% for classes B and C. The recognition effect of class C is not high performance on these two methods. One possible reason is that the training datasets for class C (motorboats, sailboats, pilot boats) are small and undertrained. Moreover, the radiation noise generated by class C is small, and it is easy to be mixed into the background noise, and the average accuracy of the two methods is low. Overall, AResnet outperforms ResNet18 in recognition.

In order to evaluate the robustness, standard deviation (STD) and arithmetic mean are used to characterize the robustness of the model. The lowest STD reflects the strong robustness of the method. The calculation formula is as follows:

$$\{\begin{array}{c} \bar{c}=\frac{\sum_{i=1}^{n}c_{i}}{n}\\ std=\sqrt{\frac{1}{n-1}\overset{n}{\underset{i=1}{\sum }}{\left(c_{i}-\bar{c}\right)}^{2}} \end{array}$$

The statistical results of the mean and variance of AResnet and ResNet18 listed in Table 6. The results show that the AResnet method achieves the minimum STD, which indicates that the AResnet method is more robust than the ResNet18 method.

We will perform five-fold cross-validation on ShipsEar, and then perform paired t-tests on the results of each fold cross-validation to show the statistical significance of the classification results obtained by the methods used in this paper. Table 7 shows the AResnet and ResNet18 t-test results for 3D fusion features. It can be observed that the AResnet method is more significant than Resnet 18 method.

### 4.3. Comparison in Computational Efficiency and Floating-Point Operation

To calculate the total calculation time of a single era of the network, the time required for forward and backward transmission of a single batch is calculated as follows:

$$t_{b}=\overset{n}{\underset{i=0}{\sum }}b\left(L_{i}\right)$$

where *n* is the number of layers in the network, and $b\left(L_{i}\right)$ is the layer *i* and $L_{i}$ belongs to the type of layer I. The total execution time of the network is calculated as follows:

$$t=omt_{b}$$

where *m* is the number of batches required to process data, and *o* is the number of cycles required to train the network.

The Table 8 shows the computational efficiency of all methods used in this study in terms of the number of floating-point operations (FLOP) and the computational time spent in each period of the model. The results in Table 8 were obtained using a system containing Nvidia GeForce RTX 3060 and AMD R7-5800H CPUs. It can be seen that the number of FLOPs of the proposed method AResnet is far more than the models based on CRNN, CNN, DNN, and ResNet18. In terms of calculation time, the proposed method also takes more time than the models based on CRNN, CNN, DNN, and ResNet18. Compared with other models, AResnet has no advantage in computing floating point numbers and computing time.

## 5. Conclusions

We propose a residual network underwater acoustic identification method based on attention, which performs better on different ship-radiated noise data sets. By increasing the width of the model and combining it with the channel attention module, we can fully extract the features of different samples and enhance the differences in the features of different channels so as to suppress the interference of ocean background noise and improve the applicability of the model in different environments. At the same time, the joint loss function, which includes the cross-entropy loss function and central loss function, monitors different features differently, solving the problem of equal monitoring of all features, including interference information, when mining the distinguishing information of different sample features. The recognition accuracy of this method on the DeepShip dataset is 99.0%. The AResnet trained on DeepShip is used as a migration learning framework. After fine-tuning, it is applied to the ShipsEar dataset. The average recognition rate reaches 98.0%, which is better than the comparison method.

The residual network based on attention focuses on target characteristics, suppresses multi-source interference, avoids gradient disappearance or gradient explosion, and can show good performance when the training data are sufficient. The residual network based on transfer learning and attention has a wide range of applications, shows good performance in underwater acoustic target recognition in different environments, and can be easily applied to other marine target recognition tasks. As part of future work, we intend to adaptively increase or decrease the number of hidden nodes during training to adapt to changing marine environments with fewer parameters.

## Figures and Tables

**Table 1:** DeepShip data recognition details.

Category	Class A	Class B	Class C	Class D
Ship type	Tug	Cargo ship	Passenger ship	Oil tanker
Total Recordings	70	110	193	240

**Table 2:** ShipsEar data recognition details.

Category	Ship Type	Total Recordings
Class A	Fishing boats, trawlers, mussel boats, tugboats, and dredgers	17
Class B	Natural environment noise	12
Class C	Motor boats, sailboats, pilot boats	19
Class D	Passenger ferry	30
Class E	Ocean-going ships and ro-ro ships	12

**Table 3:** Test results of AResnet on DeepShip dataset.

	Precision	Recall	F1-Score	Support
Class A	0.993	0.996	0.994	1695
Class B	0.982	0.966	0.974	500
Class C	0.995	0.989	0.992	1690
Class D	0.985	0.992	0.989	1690
Average	0.990	0.990	0.990	5575

**Table 4:** Test results of fusion features in different models.

	Precision	Recall	F1-Score	Support
AResnet	0.990	0.990	0.990	5575
DNN	0.980	0.980	0.980	5575
CNN	0.964	0.964	0.964	5575
CRNN	0.986	0.986	0.986	5575

**Table 5:** Test results of AResnet and ResNet18.

	Class	Precision	Recall	F1-Score	Support
AResnet	Class A	0.983	0.962	0.973	185
	Class B	1.000	0.991	0.996	115
	Class C	0.966	0.953	0.960	150
	Class D	0.981	0.981	0.981	420
	Class E	0.976	1.000	0.988	240
	Average	0.980	0.980	0.980	1110
ResNet18	Class A	0.950	0.919	0.934	185
	Class B	0.983	1.000	0.991	115
	Class C	0.909	0.867	0.887	150
	Class D	0.941	0.945	0.943	420
	Class E	0.948	0.983	0.965	240
	Average	0.944	0.944	0.944	1110

**Table 6:** Mean and STD of AResnet and ResNet18.

Method	Mean	STD
AResnet	0.981	0.012
ResNet18	0.946	0.026

**Table 7:** The results of paired t-test of AResnet and ResNet18.

Method 1	Method 2	*p*-Value (*p* < 0.05)
AResnet	ResNet18	0.0115

**Table 8:** The performance of different methods on DeepShip and ShipEar datasets.

Dataset	Method	FLOPs (G)	Params (M)	Computation Time (s/epoch)
DeepShip	AResnet	1.46 G	9.47 M	138 s
	DNN	0.02 G	17.91 M	7 s
	CNN	0.72 G	132.44 M	78 s
	CRNN	0.23 G	2.87 M	31 s
ShipsEar	AResnet	1.46 G	9.47 M	28 s
	ResNet18	0.06 G	0.78 M	7 s

## Data Availability

The data used to support the findings of this study are available from the corresponding author upon request.
